# Gaining Insights into the Codon Usage Patterns of *TP53* Gene across Eight Mammalian Species

**DOI:** 10.1371/journal.pone.0121709

**Published:** 2015-03-25

**Authors:** Tarikul Huda Mazumder, Supriyo Chakraborty

**Affiliations:** Department of Biotechnology, Assam University, Silchar-788011, Assam, India; Centers for Disease Control and Prevention, UNITED STATES

## Abstract

*TP53* gene is known as the “guardian of the genome” as it plays a vital role in regulating cell cycle, cell proliferation, DNA damage repair, initiation of programmed cell death and suppressing tumor growth. Non uniform usage of synonymous codons for a specific amino acid during translation of protein known as codon usage bias (CUB) is a unique property of the genome and shows species specific deviation. Analysis of codon usage bias with compositional dynamics of coding sequences has contributed to the better understanding of the molecular mechanism and the evolution of a particular gene. In this study, the complete nucleotide coding sequences of *TP53* gene from eight different mammalian species were used for CUB analysis. Our results showed that the codon usage patterns in *TP53* gene across different mammalian species has been influenced by GC bias particularly GC_3_ and a moderate bias exists in the codon usage of *TP53* gene. Moreover, we observed that nature has highly favored the most over represented codon CTG for leucine amino acid but selected against the ATA codon for isoleucine in *TP53* gene across all mammalian species during the course of evolution.

## Introduction

*TP53* gene encodes tumor protein p53 which is known as the “guardian of the genome” as it plays a vital role in maintaining genomic stability by preventing mutation in the genome [1]. The p53 primarily acts as transcription factor and stands out as a key player in restricting tumor cell invasion that includes the ability to induce cell cycle arrest, DNA repair, senescence and apoptosis [2]. Mutation in p53 results in abnormal proliferation of cells that leads to the formation of tumor development and so *TP53* gene is cataloged as tumor suppressor gene [3].

The nucleus of a cell is the main store house of tumor protein p53 where it binds to DNA. When any damage occurs in the DNA of a cell by some external agents like toxic chemicals, radiation, exposure to sun light or ultra violet rays, p53 plays the crucial role in activating other genes and inhibits cell cycle to repair the damage [4]. In case of failure of DNA repair, the tumor protein p53 prevents the cell from dividing and provokes signals to a wide variety of genes that contribute to TP53 mediated cell death *i*.*e*., apoptosis [5].

Unequal usage of synonymous codons that encode the same amino acid during translation of a gene into protein is known as codon usage bias (CUB). Some codons in a synonymous group are used more frequently whereas others less frequently in the genome of an organism [6,7]. CUB is a unique property of the genome and it may vary between genes from the same genome or within a single gene [8,9].

The advent of whole genome sequencing in different organisms and the easily accessible nucleotide database from NCBI (GenBank) have attracted much attention of the scientific community to study CUB in gaining clues for understanding the molecular evolution of genes and genome characterization.

Previously, several studies were conducted on synonymous codon usage bias in a wide variety of organisms including prokaryotes and eukaryotes [10–16], and till date in many organisms the codon usage patterns have been interpreted for diverse reasons. Many genomic factors such as gene length, GC-content, recombination rate, gene expression level, or modulation in the genetic code are associated with CUB in different organisms [17–21]. In general, compositional constraints under natural selection or mutation pressure are considered as major factors in the codon usage variation among different organisms [8,22–25]. Moreover, studies revealed that mutation pressure, natural or translational selection, secondary protein structure, replication and selective transcription, hydrophobicity and hydrophilicity of the protein and the external environment play a major role in the codon usage pattern of organisms [26]. In unicellular and multicellular organisms it was observed that, preferred synonymous codons/optimal codons with abundant tRNA gene copy number rise with gene expression level within the genome that supports selection on high codon bias confirmed by positive correlation between optimal codons and tRNA abundance [18,22,27]. Urrutia and Hurst (2003) reported weak correlation between gene expression level and codon usage bias within human genome though not related with tRNA abundance [19]. However, Comeron (2004) observed that in human genome, highly expressed genes have preference towards codon bias favoring codons with most abundant tRNA gene copy number compared to less highly expressed genes [28].

The study of codon usage bias acquires significance in biology not only in the context of understanding the process of evolution at molecular level but also in designing transgenes for increased expression, discovering new genes [29] based on nucleotide compositional dynamics, detecting lateral gene transfer and for analyzing the functional conservation of gene expression [30]. Codon usage bias may be superimposed on the effect of natural selection. The amount of protein produced from the mRNA transcript may vary significantly since the translational properties of alternate synonymous codons are not equivalent [31]. Several studies have further shown that codon usage bias is associated with highly expressed genes as some codons are used more often than others in the coding sequences [32]. Moreover, literature suggested that a gene can be epitomized not only by the sequence of its amino acid but also by its codon usage patterns shaped by the balance between mutational bias and natural selection [33]. As a consequence of selection pressure within a gene, differentiation in codon bias may arise between species of the same genus.

The present study was undertaken in order to perform a comparative analysis of codon bias and compositional dynamics of codon usage patterns in *TP53* gene across eight different mammalian species using nucleotide chemistry (GC contents) and several genetic indices namely effective number of codons (*ENC*), relative synonymous codon usage (*RSCU*), and relative codon usage bias (*RCBS*) etc. Our analysis has given a novel insight into the codon usage patterns of *TP53* gene that would facilitate better understanding of the structural, functional as well as evolutionary significance of the gene among the mammalian species.

## Results and Discussion

### Codon usage patterns in *TP53* genes across mammalian species

**Fig 1:**
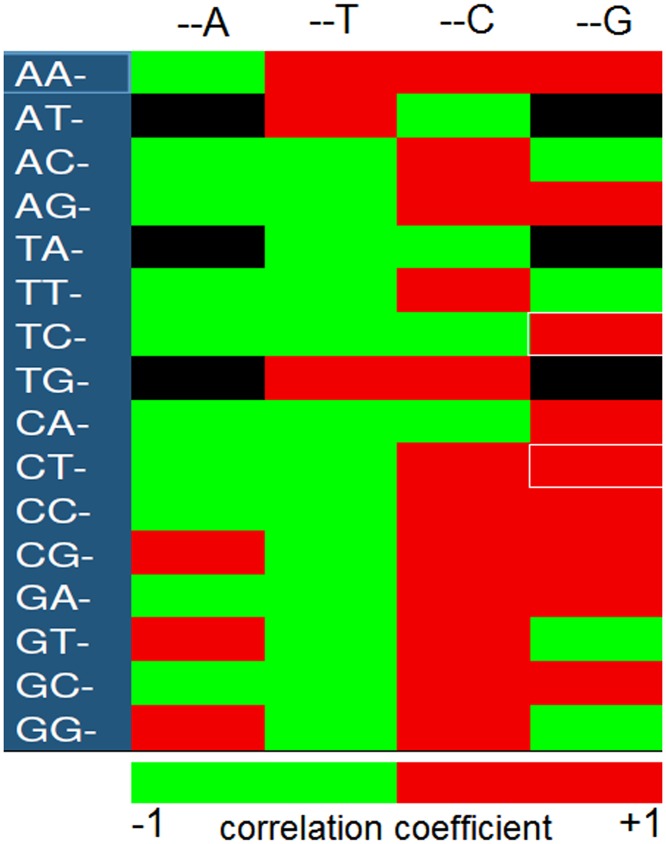
Heat maps of correlation coefficient of codons with GC_3_. The color coding red represents the positive correlation, green as negative correlation. The black fields are stop codons (TAA, TAG, TGA) and non-degenerate codons (ATG, TGG) together with ATA codons for isoleucine which was altogether absent in *TP53* gene across mammalian species. White marked rectangular boxes are the codons that showed strong positive correlation with GC_3s_.

Correlation coefficient between codon usage and GC bias was analyzed using heat map (Fig. 1) in order to find out the relationship between the codon usage variation and the GC constraints among the selected coding sequences of *TP53* genes. In our analysis, nearly all codons ending with G/C base showed positive correlation with GC bias and nearly all A/T—ending codons showed negative correlation with GC bias. But, 8 G/C—ending codons (ATC, ACG, TAC, TTG, TCC, CAC, GTG, GGG) showed negative correlation with GC bias whereas 6 A/T-ending codons (AAT, ATT, TGT, CGA, GTA, GGA) showed positive correlation with AT bias although statistically not significant (p>0.05). Two G-ending codons *i*.*e*. TCG for serine and CTG for leucine amino acid showed strong positive correlation (p<0.01) with GC_3s_, indicating that codon usage has been influenced by GC bias due to GC_3s_. Interestingly, we observed that the codon ATA encoding isoleucine amino acid was not favored by natural selection in *TP53* genes across mammalian species during the course of evolution. Thus, scanning the codon usage pattern provides the basis of the mechanism for synonymous codon usage bias and has both practical as well as theoretical significance in gaining clues of understanding molecular biology [34].

### G/C-ending codons are favored by *TP53* gene across mammalian species

**Fig 2:**
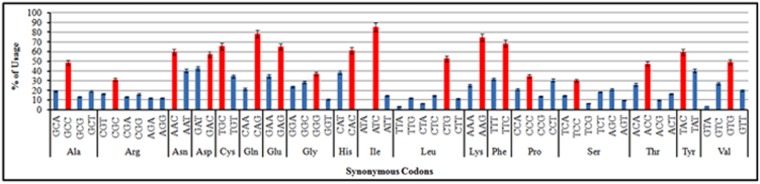
Overall frequency of optimal and non optimal codon used in *TP53* genes among mammals. Red color coding represents optimal used codons with corresponding amino acid.

We analyzed the nucleotide composition of coding sequences from *TP53* genes (Table 1) which revealed that mean value of C (361.50) was the highest followed by G (306.75), A (270.88) and T (227.50) among all the selected mammals. The mean percentage of GC and AT compositions was 57.3% and 42.7% respectively. Thus, the overall nucleotide composition suggested that the nucleotide C and G occurred more frequently compared to A and T in the coding sequences of *TP53* gene across the mammalian species. The nucleotide composition at the third position of codon (A_3_,T_3_,G_3_,C_3_) showed that the mean values of C_3_ and G_3_ were the highest followed by T_3_ and A_3_. The GC_3_ values (ranged from 58.7%-70.6%, mean = 65.1%, SD = 0.040) was compared with that of AT_3_ values (ranged from 29.4%-41.3%, mean = 34.9%, SD = 0.040) in the coding sequences of *TP53* genes. The average percentage of GC contents at the first and second codon positions (GC_12_) was found in the range of 52.6% to 54.9% with a mean value of 53.4% and a standard deviation (SD) of 0.008. Therefore, nucleotide composition analysis suggested that GC—ending codons might be preferred over AT—ending codons in the coding sequences of *TP53* genes across the selected mammalian species. Further, we calculated the occurrence of frequently used optimal codons (Fop) for each amino acid as suggested by Lavner and Kotler (2005) [14]. The frequency was allied with statistical analysis to find out the highest and lowest frequently used codon. Our results showed that the most frequently used codons were G/C—ending for the corresponding amino acid (Fig. 2) in *TP53* genes across mammalian species.

**Table 1:** Nucleotide composition analysis in the coding sequences of *TP53* gene.

Sl.	A	T	G	C	A_3_	T_3_	G_3_	C_3_	AT	GC	GC_1_	GC_2_	GC_3_	AT_3_	GC_12_
No.									%	%	%	%	%	%	%
1	262	224	308	358	55	69	122	138	42.2	57.8	56.8	49.0	67.7	32.3	52.9
2	267	234	294	366	56	79	117	135	43.2	56.8	56.8	48.6	65.1	34.9	52.7
3	261	219	321	360	53	66	134	134	41.3	58.7	57.4	49.4	69.3	30.7	53.4
4	266	229	300	351	55	78	115	134	43.2	56.8	56.5	48.7	65.2	34.8	52.6
5	264	204	321	384	57	58	125	151	39.9	60.1	59.8	49.9	70.6	29.4	54.9
6	289	244	302	341	73	89	112	118	45.3	54.7	57.4	48.0	58.7	41.3	52.7
7	282	232	301	367	68	82	112	132	43.5	56.5	58.1	49.5	61.9	38.1	53.8
8	276	234	307	365	62	86	115	131	43.1	56.9	59.1	49.0	62.4	37.6	54.1
Mean	270.9	227.5	307	361.5	59.9	75.9	119	134.1	42.7	57.3	57.7	49.0	65.1	34.9	53.4
SD	10.30	12.05	9.79	12.6	7.18	10.6	7.60	9.06	0.016	0.016	0.011	0.005	0.040	0.040	0.008

SD: standard deviation, GC_12_: average of GC contents at first and second codon positions.

### Relative synonymous codon usage in *TP53* gene across mammals

**Fig 3:**
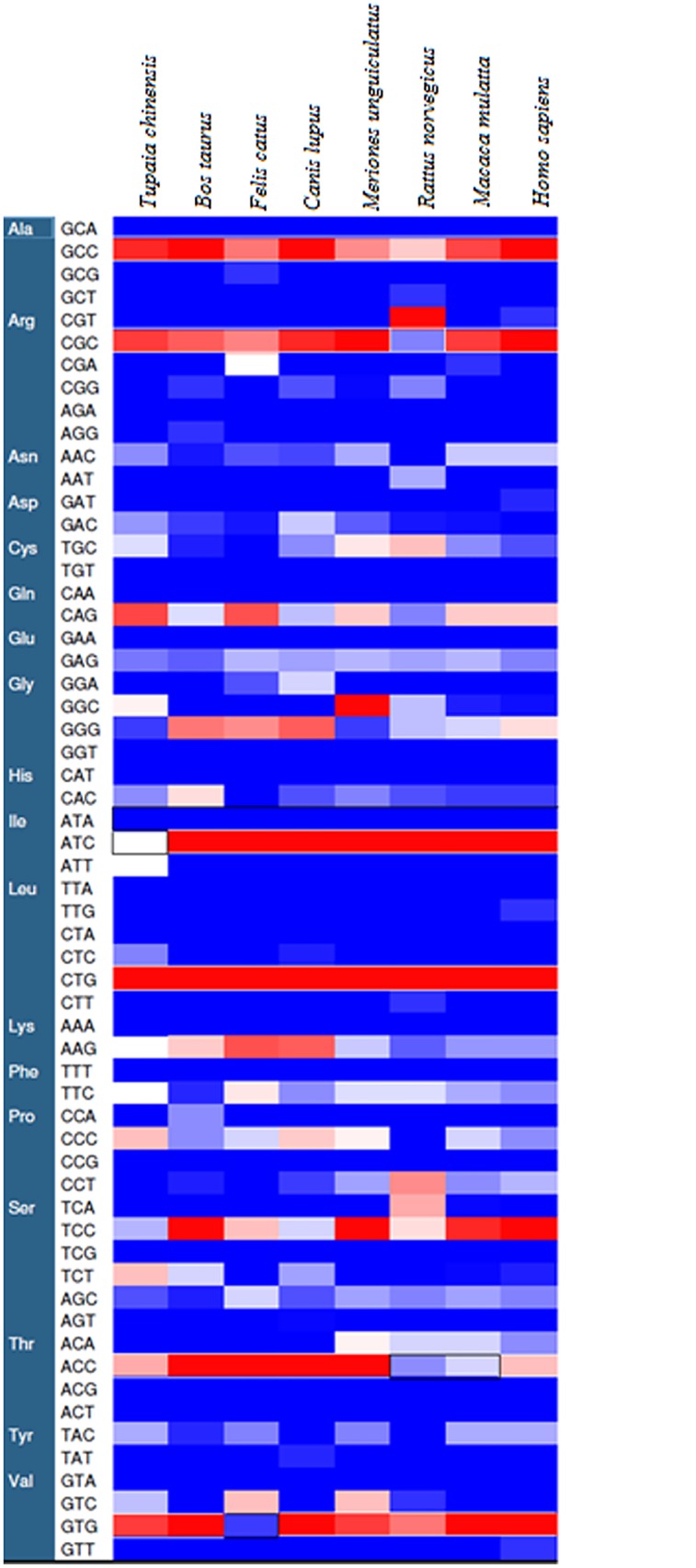
Clustering of RSCU values of each codon among *TP53* gene across mammals. Each rectangular box on the map represents the RSCU value of a codon (shown in rows) corresponding to the *TP53* gene across mammalian species (shown in columns). The intensity of color coding indicates different RSCU values: intensity towards blue RSCU<1, white RSCU>1 and red RSCU>1.6.

The relative synonymous codon usage values of 59 codons for *TP53* gene across eight mammalian species were analyzed excluding the codons ATG (methionine) and TGG (tryptophan). In our calculation RSCU value greater than 1.0 represents that the particular codon is used more frequently and less than 1.0 represents the less frequently used codon for the corresponding amino acid. The RSCU value greater than 1.6 indicates over represented codon for the corresponding amino acid. The overall RSCU values in the selected coding sequences of *TP53* gene revealed that 25 codons were most frequently used among the 59 codons and the most predominantly used codons were G/C—ending compared to A/T—ending (Table 2). Besides, it was observed that C—ending codon was mostly favored compared to G—ending codon in the coding sequence of *TP53* gene among the selected mammalian species. Our results showed marked similarities as reported by Dass *et al*., (2012) in serotonin receptor gene family from different mammalian species [35]. Further, clustering analysis of RSCU values (Fig. 3) depicted that the codon GCC, CGC (except *Rattus norvegicus*), ATC (except *Tupaia chinensis*), CTG, ACC (except *Rattus norvegicus*, *Macaca mulatta*), GTG (except *Felis catus*) were displayed as the over represented codons (RSCU>1.6). The highest RSCU value was found for the codon CTG for leucine amino acid in all *TP53* genes across mammalian species. The codon ATA showed the RSCU value zero because natural selection has not favored this codon in *TP53* gene across mammalian species.

**Table 2:** Overall relative synonymous codon usage patterns (RSCU) for *TP53* gene among eight mammalian species.

Amino Acid	Codon	N	RSCU^a^	Amino Acid	Codon	N	RSCU^a^
Ala	GCA	37	0.78	Leu	TTA	9	0.2
	GCC*	91	1.95		TTG	31	0.71
	GCG	26	0.53		CTA	17	0.38
	GCT	36	0.76		CTC	39	0.88
Arg	CGT	34	0.98		CTG*	143	3.18
	CGC*	65	1.86		CTT	30	0.68
	CGA	27	0.78	Lys	AAA	42	0.51
	CGG	33	0.96		AAG*	125	1.5
	AGA	24	0.71	Phe	TTT	29	0.63
	AGG	25	0.73		TTC*	61	1.37
Asn	AAC*	68	1.2	Pro	CCA	70	0.84
	AAT	44	0.81		CCC*	115	1.4
Asp	GAT	67	0.86		CCG	44	0.55
	GAC*	82	1.15		CCT*	100	1.22
Cys	TGC*	60	1.31	Ser	TCA	45	0.86
	TGT	32	0.69		TCC*	94	1.81
Gln	CAA	23	0.43		TCG	21	0.4
	CAG*	82	1.57		TCT*	58	1.09
Glu	GAA	84	0.7		AGC*	66	1.26
	GAG*	157	1.31		AGT*	30	0.57
Gly	GGA	41	0.95	Thr	ACA*	47	1.05
	GGC*	49	1.13		ACC*	84	1.91
	GGG*	65	1.5		ACG	18	0.4
	GGT	18	0.43		ACT	29	0.65
His	CAT	32	0.78	Tyr	TAC*	45	1.19
	CAC*	50	1.23		TAT	32	0.81
Ile	ATA	0	0	Val	GTA	5	0.15
	ATC*	61	2.58		GTC*	38	1.1
	ATT	11	0.43		GTG*	69	1.98
					GTT	28	0.8

^a^ mean values of RSCU based on the synonymous codon usage frequencies of *TP53* gene, N: Total number of preferred codons,

*RSCU>1.

### Codon usage patterns of *TP53* gene correspond to phylogeny of mammalian species

**Fig 4:**
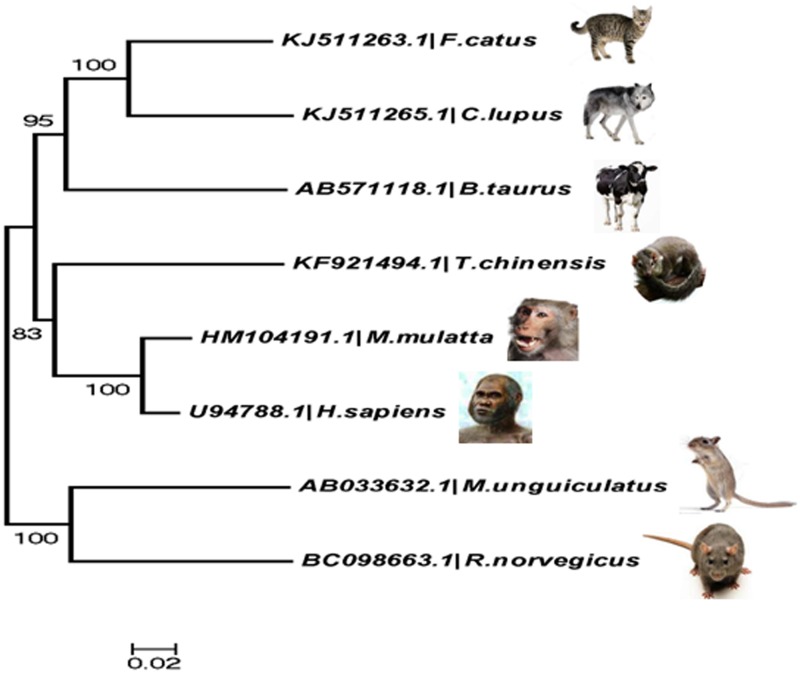
Phylogenetic analysis of the Kimura 2- parameter (K2P) distances of the selected coding sequences among *TP53* genes of different mammalian species. The percentage of replicate trees in which the associated taxa clustered together in the bootstrap test (1000 replicates) is shown next to the branches. The tree is drawn to scale, with branch lengths in the same units as those of the evolutionary distances used to infer the phylogenetic tree. The evolutionary distances were computed using the Kimura 2-parameter method and are in the units of the number of base substitutions per site. The analysis involved 8 cds sequences. All positions containing gaps and missing data were eliminated. There were a total of 1200 positions in the final dataset. Evolutionary analyses were performed in MEGA6.

We have performed a neighbor joining tree analysis based on Kimura 2-parameter (K2P) distances of the coding sequences in *TP53* gene across mammalian species (Fig. 4). We observed that codon usage patterns in *TP53* genes have significant similarities among the closely related mammalian species. The gene *TP53* in *H*. *sapiens* showed resemblance to the *TP53* gene in *M*. *mulatta*, Similarly, *TP53* of *F*. *catus* resembled to that of *C*. *lupus* and *M*.*unguiculatus* with *R*. *norvigicus*. Generally, genes with similar functions exhibit similar patterns of codon usage frequency [36]. Our analysis further suggested that the coding sequence of *TP53* gene share similar patterns of codon usage bias across eight mammalian species.

### Selection pressure over *TP53* gene across mammalian species

**Fig 5:**
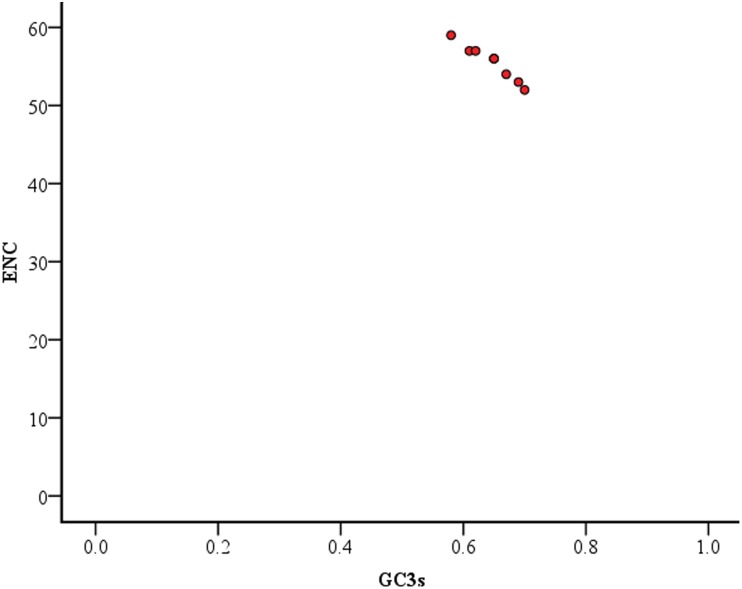
ENC vs GC3s values for *TP53* gene. Red dots represent the ENC and GC_3s_ values of the coding sequences for *TP53* gene across mammalian species.

The ENC values of the coding sequences ranged from 52 to 59 with a mean of 55.5±2.33 indicating relatively smaller variation in the codon usage of *TP53* gene across eight mammalian species. However, the GC_3s_ values ranged from 0.59 to 0.71 with a mean value of 0.65±0.040. Significant negative correlation (Pearson r = -0.979, p<0.01) was observed between ENC and GC_3s_. Moreover, a plot of ENC *vs* GC_3s_ revealed that the ENC values had negative correlation with the GC_3_ content (Fig. 5) and comparatively lower ENC was linked to higher GC_3s_ values. All the selected coding sequences of *TP53* gene across the selected mammalian species had a higher predominance of G/C—ending codons. It suggested that GC_3s_ values determined the codon usage pattern in the coding sequences of *TP53* gene [33]. Nabiyouni *et al*., (2013) reported that eukaryotic organisms with very high GC-contents have high GC_3_-composition while organisms with low GC-content have low GC_3_-composition in the genome [37]. We also calculated GC_3_ skew values which ranged from 0.000 to -0.094, indicating that GC_3_ composition at the third position of codon might have played an important role in the codon usage bias [38]. Negative GC skew was observed in all the coding sequences of *TP53* gene which revealed that the abundance of C over G [39]. In addition, lower values of the frequency of optimal codons (FOP) and the effective number of codons (ENC) along with higher GC contents suggested that a moderate bias exists in the usage of synonymous codons [33] for *TP53* gene in different mammalian species. Predominant codon usage bias was observed in *TP53* gene of *M*.*unguiculatus* compared to other mammalian species (Table 3).

**Table 3:** Codon usage bias indices for *TP53* gene across mammalian species.

MAMMALS	RCBS	ENC	GC_3s_	FOP	Highest RSCU	GC Skew	GC_3_ Skew
*Tupaia chinensis*	0.006	54	0.68	0.305	CTG (Leu)	-0.08	0.061
*Bos taurus*	0.015	56	0.65	0.305	CTG (Leu)	-0.11	0.071
*Felis catus*	0.03	53	0.69	0.305	CTG (Leu)	-0.06	0
*Canis lupus*	0.042	56	0.65	0.305	CTG (Leu)	-0.08	0.076
*Meriones unguiculatus*	0.043	52	0.71	0.306	CTG (Leu)	-0.09	0.094
*Rattus norvegicus*	0.054	59	0.59	0.306	CTG (Leu)	-0.06	0.026
*Macaca mulatta*	0.063	57	0.62	0.305	CTG (Leu)	-0.10	0.081
*Homo sapiens*	0.065	57	0.62	0.305	CTG (Leu)	-0.09	0.065

RCBS-Relative codon usage bias, ENC-Effective number of codons, GC_3s_-GC contents at third positions of codon, FOP-Frequency of optimal codons, RSCU-Relative synonymous codon usage.

**Fig 6:**
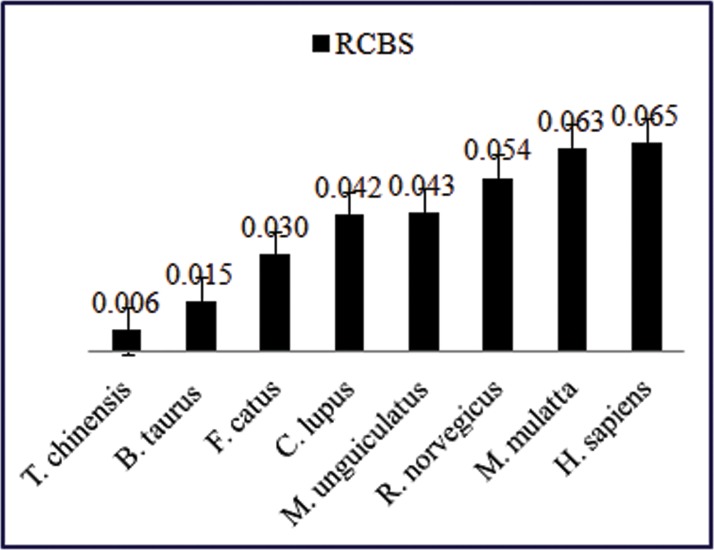
Distribution of RCBS for *TP53* gene across eight mammalian species.

*RCBS* value of a gene can be used as an effective measure of predicting gene expression and its value depends on the patterns of codon usage along with nucleotide compositional bias of a gene [20]. The distribution of *RCBS* values for *TP53* gene across eight mammalian species is shown in figure below (Fig. 6). The *RCBS* values ranged from 0.006 to 0.065 with a mean value of 0.039 and a standard deviation (SD) of 0.021. In our analysis, low mean *RCBS* value suggested that there exists a low codon bias for *TP53* gene associated with low expression level [20].

## Conclusions

In brief, our results showed that codon usage in *TP53* gene in mammals has been influenced by GC bias, mainly due to GC_3s_. The majority of frequently used codons were G/C ending in which C—ending codons were mostly favored compared to G—ending codons for the corresponding amino acid. The most over-represented codon was CTG encoding the amino acid leucine in the *TP53* gene of all the selected mammalian species. We further observed that the codon ATA encoding isoleucine was selected against by nature in *TP53* genes across the mammalian species under study during the course of evolution. The codon usage pattern for *TP53* in *H*. *sapiens* showed resemblance to that of *M*. *mulatta*; similarly, *F*. *catus* to *C*. *lupus* and *M*. *unguiculatus* to *R*. *norvigicus*. Moderate codon bias was observed for the *TP53* gene in different mammalian species.

The codon usage patterns in the coding sequence of *TP53* gene across different mammalian species showed significant similarities, suggesting that the evolutionary pattern might be similar. According to Yang and Nielsen (2008), codon bias in mammals is mainly influenced by mutation bias and the selection on codon bias is weak for nearly neutral synonymous mutations [40]. From the outstanding work of Grantham *et al*., (1980–1981) on “genome hypothesis” it was evident that species specific genes share similar spectrum of codon usage frequency [41,42]. The present study revealed that specific gene of closely related species with similar functions exhibit similar patterns of codon bias across different mammals as evident from the previous work of Dass *et al*., (2012) [35]. To the best of our knowledge, this is the first report on the codon usage pattern in *TP53* gene across the mammalian species. Since our analysis has given better insights into the codon usage, it may have theoretical value in further understanding the molecular evolution of *TP53*gene.

## Materials and Methods

### Sequence Data

The complete nucleotide coding sequences (cds) for TP53 gene having perfect start and stop codon, devoid of any unknown bases (N) and perfect multiple of three bases, were retrieved from National Center for Biotechnology Information (NCBI) GenBank database (http://www.ncbi.nlm.nih.gov). Finally, we selected eight coding sequences for TP53 gene that fulfill the above mentioned criteria in different mammalian species and used in our CUB analysis (Table 4).

**Table 4:** Eight mammalian species with accession number and length (bp) of coding sequences for *TP53* gene.

**CDS NO.**	**MAMMALS**	**ACCESSION NO.**	**Length (bp)**
**1**	*Tupaia chinensis*	KF921494.1	1152
**2**	*Bos taurus*	AB571118.1	1161
**3**	*Felis catus*	KJ511263.1	1161
**4**	*Canis lupus*	KJ511265.1	1146
**5**	*Meriones unguiculatus*	AB033632.1	1173
**6**	*Rattus norvegicus*	BC098663.1	1176
**7**	*Macaca mulatta*	HM104191.1	1182
**8**	*Homo sapiens*	U94788.1	1182

CDS NO: Coding sequence number.

### Prediction of Base Composition Bias

The occurrence of overall frequency of the nucleotide (G+C) at first (GC_1_), second (GC_2_) and third (GC_3_) position of synonymous codons were calculated to quantify the extent of base composition bias. Moreover, we analyzed the skewness for AT, GC and GC_3s_ of each coding sequence to estimate the base composition bias particularly in relation to transcription processes.

### Effective Number of Codons (ENC) Analysis

ENC is generally used to quantify the codon usage bias of a gene that is independent of the gene length and number of amino acids [43]. This measure was computed as per Wright (1990) to estimate the extent of CUB exhibited by the coding sequences of *TP53* gene across the selected mammalian species:

$$ENC=2+\frac{9}{F_{2}}+\frac{1}{F_{3}}+\frac{5}{F_{4}}+\frac{3}{F_{6}}$$

Where, F_k_ (_k_ = 2, 3, 4 or 6) is the average of the F_k_ values for k-fold degenerate amino acids. The F value denotes the probability that two randomly chosen codons for an amino acid with two codons are identical.

The values of ENC ranged from 20 indicating strong codon bias in the gene using only one synonymous codon for the corresponding amino acid, to 61indicating no bias in the gene using all synonymous codons equally for the corresponding amino acid [43].

### Frequency of Optimal Codon (Fop) Analysis

Fop is a measure of codon usage bias in a gene [44]. Fop values represent the ratio of the number of optimal codons used to the total number of synonymous codons [22]. The Fop value ranges from 0.36 for a gene showing uniform codon usage bias to 1 for a gene showing strong codon usage bias [45]. Fop value for each selected coding sequence was calculated using the formula given by Lavner and Kotler (2005) [14].

### Relative Synonymous Codon Usage (RSCU) Analysis

RSCU is defined as the observed frequency of a codon divided by the expected frequency if all codons are used equally for any particular amino acid [46]. RSCU values of codons for each of the selected coding sequence of *TP53* gene was calculated as follows:

$$RSCU=\frac{g_{ij}}{\sum_{j}^{ni}g_{ij}}n_{i}$$

Where, *g*_*ij*_ is the observed number of the *i*th codon for the *j*th amino acid which has *n*_*i*_ kinds of synonymous codons [26].

### Computation of Gene Expression

Gene expression was estimated through RCBS which can be defined as the overall score of a gene indicating the influence of relative codon bias (RCB) of each codon in a gene [20]. The RCBS value of each coding sequence of *TP53* gene was calculated as follows:

$$w_{c}^{RCB}=\frac{O_{C}-E[O_{C}]}{E[O_{C}]}$$

where, *Oc* is the observed number of counts of codon *c* of the query sequence and *E*[*O*_*c*_] is the expected number of codon occurrences given the nucleotide distribution at three codon positions (b1b2b3) [20].

$$RCB=\exp(\frac{1}{O_{tot}}\sum_{c\in C}\log w_{c}^{RCB})-1$$

[47]

Where, *O*_***tot***_ is the total number of codons

### Software Used

The above mentioned genetic indices were estimated in a PERL program developed by SC (corresponding author) to measure the CUB on the selected coding sequences of *TP53* genes in different mammalian species. All statistical analyses were carried out using the SPSS software. Cluster analysis (Heat map) of correlation coefficient of codons with GC3 and the RSCU values of codons among the eight mammalian species were clustered using a hierarchical clustering method implemented in NetWalker software [48].

## References

[1] Lane DP (1992) Cancer. p53, guardian of the genome. Nature 358: 15–16. 1614522 10.1038/358015a0

[2] Rivlin N, Brosh R, Oren M, Rotter V (2011) Mutations in the p53 Tumor Suppressor Gene: Important Milestones at the Various Steps of Tumorigenesis. Genes Cancer 2: 466–474. 10.1177/1947601911408889 21779514 PMC3135636

[3] McBride OW, Merry D, Givol D (1986) The gene for human p53 cellular tumor antigen is located on chromosome 17 short arm (17p13). Proc Natl Acad Sci U S A 83: 130–134. 3001719 10.1073/pnas.83.1.130 PMC322805

[4] Vousden KH, Lu X (2002) Live or let die: the cell's response to p53. Nat Rev Cancer 2: 594–604. 12154352 10.1038/nrc864

[5] Carnero A, Hudson JD, Hannon GJ, Beach DH (2000) Loss-of-function genetics in mammalian cells: the p53 tumor suppressor model. Nucleic Acids Res 28: 2234–2241. 10871344 10.1093/nar/28.11.2234 PMC102629

[6] Hooper SD, Berg OG (2000) Gradients in nucleotide and codon usage along Escherichia coli genes. Nucleic Acids Res 28: 3517–3523. 10982871 10.1093/nar/28.18.3517 PMC110745

[7] Behura SK, Severson DW (2012) Comparative analysis of codon usage bias and codon context patterns between dipteran and hymenopteran sequenced genomes. PLoS One 7: e43111 10.1371/journal.pone.0043111 22912801 PMC3422295

[8] Duret L, Mouchiroud D (1999) Expression pattern and, surprisingly, gene length shape codon usage in Caenorhabditis, Drosophila, and Arabidopsis. Proc Natl Acad Sci U S A 96: 4482–4487. 10200288 10.1073/pnas.96.8.4482 PMC16358

[9] Supek F, Vlahovicek K (2005) Comparison of codon usage measures and their applicability in prediction of microbial gene expressivity. BMC Bioinformatics 6: 182 16029499 10.1186/1471-2105-6-182 PMC1199580

[10] McInerney JO (1998) Replicational and transcriptional selection on codon usage in Borrelia burgdorferi. Proc Natl Acad Sci U S A 95: 10698–10703. 9724767 10.1073/pnas.95.18.10698 PMC27958

[11] Ermolaeva MD (2001) Synonymous codon usage in bacteria. Curr Issues Mol Biol 3: 91–97. 11719972

[12] Jenkins GM, Holmes EC (2003) The extent of codon usage bias in human RNA viruses and its evolutionary origin. Virus Res 92: 1–7. 12606071 10.1016/s0168-1702(02)00309-x

[13] Gu W, Zhou T, Ma J, Sun X, Lu Z (2004) Analysis of synonymous codon usage in SARS Coronavirus and other viruses in the Nidovirales. Virus Res 101: 155–161. 15041183 10.1016/j.virusres.2004.01.006 PMC7127446

[14] Lavner Y, Kotlar D (2005) Codon bias as a factor in regulating expression via translation rate in the human genome. Gene 345: 127–138. 15716084 10.1016/j.gene.2004.11.035

[15] Liu H, Huang Y, Du X, Chen Z, Zeng X, Chen Y, et al (2012) Patterns of synonymous codon usage bias in the model grass Brachypodium distachyon. Genet Mol Res 11: 4695–4706. 10.4238/2012.October.17.3 23096921

[16] Ahn I, Jeong BJ, Son HS (2009) Comparative study of synonymous codon usage variations between the nucleocapsid and spike genes of coronavirus, and C-type lectin domain genes of human and mouse. Exp Mol Med 41: 746–756. 10.3858/emm.2009.41.10.081 19561398 PMC2772977

[17] Palidwor GA, Perkins TJ, Xia X (2010) A general model of codon bias due to GC mutational bias. PLoS One 5: e13431 10.1371/journal.pone.0013431 21048949 PMC2965080

[18] Duret L (2000) tRNA gene number and codon usage in the C. elegans genome are co-adapted for optimal translation of highly expressed genes. Trends Genet 16: 287–289. 10858656 10.1016/s0168-9525(00)02041-2

[19] Urrutia AO, Hurst LD (2003) The signature of selection mediated by expression on human genes. Genome Res 13: 2260–2264. 12975314 10.1101/gr.641103 PMC403694

[20] Roymondal U, Das S, Sahoo S (2009) Predicting gene expression level from relative codon usage bias: an application to Escherichia coli genome. DNA Res 16: 13–30. 10.1093/dnares/dsn029 19131380 PMC2646356

[21] Karlin S, Mrazek J (1996) What drives codon choices in human genes? J Mol Biol 262: 459–472. 8893856 10.1006/jmbi.1996.0528

[22] Ikemura T (1981) Correlation between the abundance of Escherichia coli transfer RNAs and the occurrence of the respective codons in its protein genes: a proposal for a synonymous codon choice that is optimal for the E. coli translational system. J Mol Biol 151: 389–409. 6175758 10.1016/0022-2836(81)90003-6

[23] Li WH (1987) Models of nearly neutral mutations with particular implications for nonrandom usage of synonymous codons. J Mol Evol 24: 337–345. 3110426 10.1007/BF02134132

[24] Xu C, Cai X, Chen Q, Zhou H, Cai Y, Ben A (2011) Factors affecting synonymous codon usage bias in chloroplast genome of oncidium gower ramsey. Evol Bioinform Online 7: 271–278. 10.4137/EBO.S8092 22253533 PMC3255522

[25] Nair RR, Nandhini MB, Sethuraman T, Doss G (2013) Mutational pressure dictates synonymous codon usage in freshwater unicellular alpha—cyanobacterial descendant Paulinella chromatophora and beta—cyanobacterium Synechococcus elongatus PCC6301. Springerplus 2: 492 10.1186/2193-1801-2-492 24255825 PMC3825069

[26] Butt AM, Nasrullah I, Tong Y (2014) Genome-wide analysis of codon usage and influencing factors in chikungunya viruses. PLoS One 9: e90905 10.1371/journal.pone.0090905 24595095 PMC3942501

[27] Akashi H (1995) Inferring weak selection from patterns of polymorphism and divergence at "silent" sites in Drosophila DNA. Genetics 139: 1067–1076. 7713409 10.1093/genetics/139.2.1067 PMC1206357

[28] Comeron JM (2004) Selective and mutational patterns associated with gene expression in humans: influences on synonymous composition and intron presence. Genetics 167: 1293–1304. 15280243 10.1534/genetics.104.026351 PMC1470943

[29] Carbone A, Zinovyev A, Kepes F (2003) Codon adaptation index as a measure of dominating codon bias. Bioinformatics 19: 2005–2015. 14594704 10.1093/bioinformatics/btg272

[30] Lithwick G, Margalit H (2005) Relative predicted protein levels of functionally associated proteins are conserved across organisms. Nucleic Acids Res 33: 1051–1057. 15718304 10.1093/nar/gki261 PMC549420

[31] Miyasaka H (2002) Translation initiation AUG context varies with codon usage bias and gene length in Drosophila melanogaster. J Mol Evol 55: 52–64. 12165842 10.1007/s00239-001-0090-1

[32] Sueoka N (1988) Directional mutation pressure and neutral molecular evolution. Proc Natl Acad Sci U S A 85: 2653–2657. 3357886 10.1073/pnas.85.8.2653 PMC280056

[33] Mandlik V, Shinde S, Singh S (2014) Molecular evolution of the enzymes involved in the sphingolipid metabolism of Leishmania: selection pressure in relation to functional divergence and conservation. BMC Evol Biol 14: 142 10.1186/1471-2148-14-142 24951280 PMC4092354

[34] Hassan S, Mahalingam V, Kumar V (2009) Synonymous codon usage analysis of thirty two mycobacteriophage genomes. Adv Bioinformatics: 316936 10.1155/2009/316936 20150956 PMC2817497

[35] Dass JF, Sudandiradoss C (2012) Insight into pattern of codon biasness and nucleotide base usage in serotonin receptor gene family from different mammalian species. Gene 503: 92–100. 10.1016/j.gene.2012.03.057 22480817

[36] Ma J, Zhou T, Gu W, Sun X, Lu Z (2002) Cluster analysis of the codon use frequency of MHC genes from different species. Biosystems 65: 199–207. 12069729 10.1016/s0303-2647(02)00016-3

[37] Nabiyouni M, Prakash A, Fedorov A (2013) Vertebrate codon bias indicates a highly GC-rich ancestral genome. Gene 519: 113–119. 10.1016/j.gene.2013.01.033 23376453

[38] Tatarinova TV, Alexandrov NN, Bouck JB, Feldmann KA (2010) GC3 biology in corn, rice, sorghum and other grasses. BMC Genomics 11: 308 10.1186/1471-2164-11-308 20470436 PMC2895627

[39] Tillier ER, Collins RA (2000) The contributions of replication orientation, gene direction, and signal sequences to base-composition asymmetries in bacterial genomes. J Mol Evol 50: 249–257. 10754068 10.1007/s002399910029

[40] Yang Z, Nielsen R (2008) Mutation-selection models of codon substitution and their use to estimate selective strengths on codon usage. Mol Biol Evol 25: 568–579. 10.1093/molbev/msm284 18178545

[41] Grantham R, Gautier C, Gouy M, Mercier R, Pave A (1980) Codon catalog usage and the genome hypothesis. Nucleic Acids Res 8: r49–r62. 6986610 10.1093/nar/8.1.197-c PMC327256

[42] Grantham R, Gautier C, Gouy M, Jacobzone M, Mercier R (1981) Codon catalog usage is a genome strategy modulated for gene expressivity. Nucleic Acids Res 9: r43–74. 7208352 10.1093/nar/9.1.213-b PMC326682

[43] Wright F (1990) The 'effective number of codons' used in a gene. Gene 87: 23–29. 2110097 10.1016/0378-1119(90)90491-9

[44] Ikemura T (1985) Codon usage and tRNA content in unicellular and multicellular organisms. Mol Biol Evol 2: 13–34. 3916708 10.1093/oxfordjournals.molbev.a040335

[45] Stenico M, Lloyd AT, Sharp PM (1994) Codon usage in Caenorhabditis elegans: delineation of translational selection and mutational biases. Nucleic Acids Res 22: 2437–2446. 8041603 10.1093/nar/22.13.2437 PMC308193

[46] Sharp PM, Li WH (1986) Codon usage in regulatory genes in Escherichia coli does not reflect selection for 'rare' codons. Nucleic Acids Res 14: 7737–7749. 3534792 10.1093/nar/14.19.7737 PMC311793

[47] Fox JM, Erill I (2010) Relative codon adaptation: a generic codon bias index for prediction of gene expression. DNA Res 17: 185–196. 10.1093/dnares/dsq012 20453079 PMC2885275

[48] Komurov K, Dursun S, Erdin S, Ram PT (2012) NetWalker: a contextual network analysis tool for functional genomics. BMC Genomics 13: 282 10.1186/1471-2164-13-282 22732065 PMC3439272

